# Trachoma

**Published:** 1908-10-31

**Authors:** Reginald E. Bickerton

**Affiliations:** Asst. Ophthalmic Surgeon, Seamen's Hospital; Lecturer on Practical Ophthalmology at the London Polyclinic


### Hospital Clinics.

### TRACHOMA.

By REGINALD E. BICKERTON, M.B., Ch.B., Asst. Ophthalmic Surgeon, Seamen's Hospital; Lecturer on Practical Ophthalmology at the London Polyclinic.

(Abstract of a Lecture delivered at the Polyclinic.)

Trachoma is an infective inflammation of the conjunctiva of the lids, characterised by extreme roughness and great hypertrophy. In its early stages trachoma is not infrequently overlooked, being mistaken for an ordinary conjunctival catarrh or follicular conjunctivitis. The risk in such circumstances is not so much to the patient himself, as to others who may come into contact with him, as in schools, barracks, etc. This hypertrophy of the conjunctiva lining the lids, gives rise, by its subsequent scarring and contraction, to the many deformities of the eyelids, and misplacement of the lashes which are among the sequelae of trachoma.

The disease is also designated granular conjunctivitis; granular lids; Egyptian ophthalmia; and in the older days, contagious ophthalmia. It originates exclusively in infection from patients already affected with it. It is only directly infective through the discharge from the conjunctiva; hence the more profuse the discharge, the greater the risk of further infection. In Egypt the infection appears to be carried by the flies, which cling to the corners of the eyes of a trachomatous person, and the fact that flies in Egypt are looked upon as sacred, no doubt tends to spread the infection more readily, since little attempt is made to brush them away. The result is that trachoma is very rife amongst the natives there.

Trachoma is characterised by extreme chronicity, and may continue throughout the whole of a patient's life, or only for a few weeks. In its chronic form it is most destructive to vision, and brings about the more serious deformities met with in the eyelids. It is found most frequently in barracks, workhouses, schools, etc., where large numbers of people are crowded together in unhygienic circumstances; in schools the infection is carried by direct contact, or by the use of towels in common. It is most frequently met with in Arabia and Egypt, which may be looked upon as its original homes. In low-lying lands, such as Belgium, Holland, the region of the lower Danube, and Hungary, it is more prevalent than in other parts of Europe. High countries, such as Switzerland and the Tyrol, are almost free from it, except for imported cases. Russian and Polish Jews are great sufferers, and, moreover, constant carriers of infection all over the world. The Chinese, Japanese, and native races of America also suffer very considerably.

Trachoma is supposed to have been brought over from Egypt into Europe by the men of Napoleon's army in 1798. It appears, however, to have existed in Europe in the time of Celsius, and scarification of the conjunctiva was largely employed for its relief. During the period 1815 to 1839, it positively scourged the European armies. In 1818 in the English army 5,000 men were partly blinded by it, and within the above period 76,800 men in the Russian army were afflicted. In the Belgian army so many men had trachoma that the army became incapacitated, and the authorities, in despair, applied to a noted ophthalmologist named Jünken, then living in Berlin, to know what was to be done. He made the fatal mistake of advising that they should all be sent to their homes, and in consequence, the malady became more universally spread in Belgium than in any other European state. Ships' crews were attacked by it and rendered almost incapable of working their vessels; this was notably the case on board a French slave ship named *Le Rôdeur* in 1819, when all the slaves and the crew, with the exception of one man were partly blinded by it, and the vessel remained helpless for several days, but ultimately reached port in safety, thanks to the one unafflicted sailor, who himself contracted the disease three days after landing.

### Symptoms.

Swelling and heaviness of the eyelids; ptosis; much redness and thickening of the conjunctiva, which becomes exceedingly rough; constant discharge, which may occasionally become profuse; these are the chief symptoms. Later, the cornea becomes affected by the extension over it from above downwards, of so-called "pannus." Occasionally corneal ulcers develop, usually in the upper zone where the cornea is scraped by the roughened upper lid. Later still, entropion or ectropion of the lids may take place with displacement of the lashes, "trichiasis." In many cases patients are quite unaware of the disease until the vision becomes affected by pannus spreading over the cornea opposite the pupil, and so blocking direct vision. The essential change is hypertrophy of the conjunctiva, by which the folds of conjunctiva, where it is reflected from the globe to the lids, become much increased in extent and thickness and protrude greatly when the lids are everted. Over the tarsus, where the conjunctiva is firmly bound down to the tarsal cartilage, this thickening of the conjunctiva does not take place till late.

Two distinct forms of hypertrophy are met with: the papillary form, in which the conjunctiva presents, on everting the lids, a velvety appearance; and the granular, in which the conjunctiva becomes studded with true trachoma granules, these much more noticeable than the papillæ, and fewer in number. In the first form the papillæ may be fine, like velvet, or coarsely granular, and may present raspberry-like projections. The subjacent Meibomian glands are no longer visible. In the second form the swollen conjunctiva is studded over with grey, translucent, rounded granules, which have been likened to frog-spawn, or boiled sago; these are found in greater numbers in the retro-tarsal folds (the superior retro-tarsal folds are shown best by first everting the upper lid, then pressing the lid down behind the tarsus with the point of a glass rod), where they attain their greatest size and fullest development. These same granules in the conjunctiva over the tarsus, where the conjunctiva is firmly bound down, at first appear as small bright yellowish points only slightly raised, but later they become more protuberant and more spread out. This appearance of the everted tarsus studded with small bright, yellowish points is quite diagnostic of trachoma, even although the other symptoms of conjunctivitis may be quite slight. As a rule both forms occur together, and probably in every case of trachoma the granules appear sooner or later.

The granules themselves, however large and prominent they may be, contain simply a mass of epithelioid cells with a delicate connective tissue structure in which appear very large cells (phagocytes); when burst by pressure, they extrude their contents readily and collapse, leaving small pits. The conjunctiva of the globe rarely becomes affected, but at times the conjunctiva just at the limbus, and a millimetre or so behind it, develops a mass of modified trachomatous tissue when pannus is commencing. The great hypertrophy of the conjunctiva in the fornices subsequently gives rise to scarring and contraction, which results in closer application of the lids to the eyeball; hence, if the conjunctiva is very rough, the cornea suffers greatly from friction.

*Pannus* is a deposit of newly-formed, gelatinous, vascular tissue at the limbus, which slowly descends over the cornea, usually over that part of the cornea covered by the upper lid. It becomes very vascular, for vessels run into it from the conjunctiva and branch in an arborescent manner. Sometimes it is quite slight—a mere film—but at others it is a fleshy-looking mass of considerable thickness. In either case if it extends downwards as far as the pupil, vision will become greatly impaired. Ulcers of the cornea are frequently met with at the lower edge of a pannus. Iritis occasionally occurs as an accompaniment of pannus, and is at times difficult to make out on account of the haze and roughness of the cornea. Pannus clears up when the hypertrophy and roughness of the conjunctiva become less, though as a rule it is a very long time before the cornea clears, and a still longer time before the vessels disappear; in fact the latter may still be made out years after with the aid of a corneal magnifyer, or by the ophthalmoscopic mirror supplemented with a +20 lens, against the dilated pupil.

### Sequelæ of Trachoma.

Deformities of the eyelids, such as entropion, ectropion, and trichiasis, are amongst the most frequent sequelæ. Narrowing of the palpebral fissure and partial adherence of the lids to the globe (symblepharon) occur less frequently. Corneal nebulæ left by deep ulcers may occur in even the mildest of cases, much more so in the very chronic ones. A very fine but distinct milkiness of the cornea appears in some cases, without there having been ulceration of the cornea; this gives rise to irregular astigmatism afterwards and impaired vision, which cannot be improved with glasses. Another sequela—happily much rarer—is xerosis of the conjunctiva and cornea, due to the glands which exist in the conjunctiva, and which secrete an oily substance for the purpose of lubricating the movements of the lids, having been destroyed. As a result the conjunctiva becomes dry in patches, and to these patches neither the tears nor any other secretion will adhere. This finally extends to the whole cornea, which becomes lustreless, insensitive, dry, and parchment-like, and tortures the patient with an irritating sense of dryness of the eye which is quite intolerable. Such patients suffer from defective vision, and from night-blindness of a very marked degree. The affection is termed xerophthalmia, and is in all probability the worst conceivable termination to a case of trachoma, short of the absolute loss of both eyes.

Entropion is caused by the scarring and subsequent contraction of the conjunctiva, which exaggerates the normal curvature of the lids. The tarsal cartilage itself participates in the inflammation and hypertrophy of the overlying conjunctiva, and its subsequent contraction tends to bend it upon itself. In this manner also trichiasis is brought about; the sharp posterior margin of the lid, which normally fits closely to the globe, becomes rounded off, loses its sharp margin, and thus brings the eyelashes closer to the globe, and later turns them right on to it. Ectropion is caused by the excessive hypertrophy of the conjunctiva on the inner surface of the lid bending the margin outwards.

### Treatment.

In the active stage apply solid copper sulphate stick to the conjunctiva of the everted lids and fornices. Use three times a day lotio hydrarg. perchlor. (1 in 3,000, or if this causes too much reaction, 1 in 4,000), and apply ung. acid. borici between the lids night and morning. Later this ointment may be beneficially changed to the citrate of copper ointment (Cupricitrol). If the cornea is very hazy from nebulae, the result of ulceration, then the ung. hydrarg. oxidi flav. (1 per cent.) should be used. Trichiasis may be dealt with merely by epilation repeated as often as necessary, by electrolysis, or by the more radical methods of excising the margin of the lid containing the eyelash follicles, splitting the lid margin, and drawing the lip containing the hair follicles up away from the globe. Entropion and ectropion call for special operations for their relief; quite a moderate degree of ectropion will give rise to eversion of the lower punctum lachrymalis and cause lachrymation; when this happens, the patient, by continually wiping the eye in a downward direction, helps to increase this eversion of the lid margin and makes matters worse.

When the swelling and roughness of the conjunctiva are very marked, expression should be resorted to. In performing this slight operation the upper lid is first everted, and well cocainised by solid cocaine applied to the surface: the whole thickness of the everted lid is then squeezed between the limbs of either Knapp's roller forceps, or Graddy's semilunar forceps, and the forceps is then drawn down towards the everted margin, bursting the granules and squeezing out their contents together with the fluid from the œdematous conjunctiva. A solution of perchloride of mercury in glycerine (1 in 500) should be painted over the surface. This little operation should be repeatedly performed until all the granules have disappeared, and the ordinary treatment with lotion continued in the intervals.

